# Refining the Allostatic Self-Efficacy Theory of Fatigue and Depression Using Causal Inference

**DOI:** 10.3390/e26121127

**Published:** 2024-12-23

**Authors:** Alexander J. Hess, Dina von Werder, Olivia K. Harrison, Jakob Heinzle, Klaas Enno Stephan

**Affiliations:** 1 Translational Neuromodeling Unit, Institute for Biomedical Engineering, University of Zurich and ETH Zurich, 8032 Zurich, Switzerland; vonwerder@biomed.ee.ethz.ch (D.v.W.); olivia.harrison@otago.ac.nz (O.K.H.); heinzle@biomed.ee.ethz.ch (J.H.); stephan@biomed.ee.ethz.ch (K.E.S.); 2 Institute of Medical Technology, Brandenburg University of Technology Cottbus-Senftenberg, 03048 Cottbus, Germany; 3 Graduate School of Systemic Neurosciences, Ludwig-Maximilians-Universität München, 82152 Planegg-Martinsried, Germany; 4 Department of Psychology, University of Otago, Dunedin 9016, New Zealand; 5 Max Planck Institute for Metabolism Research, 50931 Cologne, Germany

**Keywords:** allostatic self-efficacy, fatigue, depression, causality, structural causal model, directed acyclic graph, *d*-separation, conditional independence, average causal effect

## Abstract

Allostatic self-efficacy (ASE) represents a computational theory of fatigue and depression. In brief, it postulates that (i) fatigue is a feeling state triggered by a metacognitive diagnosis of loss of control over bodily states (persistently elevated interoceptive surprise); and that (ii) generalization of low self-efficacy beliefs beyond bodily control induces depression. Here, we converted ASE theory into a structural causal model (SCM). This allowed identification of empirically testable hypotheses regarding causal relationships between the variables of interest. Applying conditional independence tests to questionnaire data from healthy volunteers, we sought to identify contradictions to the proposed SCM. Moreover, we estimated two causal effects proposed by ASE theory using three different methods. Our analyses identified specific aspects of the proposed SCM that were inconsistent with the available data. This enabled formulation of an updated SCM that can be tested against future data. Second, we confirmed the predicted negative average causal effect from metacognition of allostatic control to fatigue across all three different methods of estimation. Our study represents an initial attempt to refine and formalize ASE theory using methods from causal inference. Our results confirm key predictions from ASE theory but also suggest revisions which require empirical verification in future studies.

## 1. Introduction

Fatigue is a prominent symptom of major clinical significance in numerous disorders across medical disciplines [1,2]. It is fundamentally disabling for patients and profoundly affects their quality of life [3]. Fatigue is a common feature across a wide range of immunological and endocrine disorders, cancer, and neuropsychiatric diseases. In particular, it constitutes one of the core diagnostic criteria of major depression in standard psychiatric classification schemes (ICD-10 and DSM-5; Refs. [4,5]).

The clinical concept of fatigue is a heterogeneous construct, and the subjective perception of chronic fatigue needs to be distinguished from objectively observable fatiguability of cognitive and motor processes [6] as well as from tiredness, which not only differ in phenomenology but are thought to be mechanistically distinct [7]. This study focuses on subjectively perceived fatigue.

The pathophysiological mechanisms leading to fatigue are likely diverse [6]. Previous theories have focused on a variety of neurophysiological, immunological, and inflammatory processes. Unfortunately, there are no mechanistically interpretable clinical tests available for fatigue that could be used to guide individual treatment [6].

More recently, a novel perspective on fatigue has been proposed—the ‘allostatic self-efficacy’ theory (ASE; Refs. [6,7,8]). The ASE theory is based on computational concepts of brain–body interactions [7,8] which, in turn, are conceptually related to and inspired by Bayesian theories of perception (predictive coding; Ref. [9]) and action (active inference; Ref. [10]). Given these Bayesian roots, ‘beliefs’—i.e., probabilistic representations of latent variables—therefore play a central role in the ASE theory. However, in its current formulation, the theory does not explicitly comment on whether these beliefs are conscious or subconscious.

The ASE theory emphasizes the role of two cognitive processes for fatigue: interoception and metacognition. Interoception corresponds to the perception of bodily states and is of major importance for understanding determinants of mental health [11,12]. Many contemporary concepts of interoception are grounded in Bayesian theories of perception and conceptualize interoception as an inference process based on the brain’s generative model of sensory inputs from the body [8,12,13,14,15,16]. More specifically, interoception can be conceptualized as “inferences about bodily (physiological and biochemical) states that are coupled to regulatory processes which serve to control these states” [17]. Metacognition can be summarized as ‘cognition about cognition’ [18], comprising a variety of evaluation processes by which the brain monitors its own performance. Building on a generic mathematical model of brain–body interactions, the ASE theory describes how the brain attempts to control bodily states via monitoring interoceptive surprise (as an index of the degree of dyshomeostasis; Ref. [7]).

In brief, the ASE theory proposes that the subjective experience of fatigue arises when, in a situation of persistent dyshomeostasis (and thus enduringly elevated interoceptive surprise), the brain arrives at the metacognitive diagnosis that its control over bodily states is failing; a condition also referred to as low allostatic self-efficacy. Put differently, the ASE theory views fatigue as a feeling state that arises from a specific metacognitive process—i.e., the insight that regulatory actions are not capable of reducing interoceptive surprise—and which signals the imperative need to rest because active regulatory actions fail to resolve dyshomeostasis. While rest also represents a regulatory action, here, we contrast it to targeted regulatory actions that involve active behavior of an overt or latent (autonomic nervous system related) nature. The theory predicts that if rest cannot resolve dyshomeostasis and thus reduce interoceptive surprise, fatigue becomes entrenched and chronic. Furthermore, if low self-efficacy beliefs generalize beyond the body, leading to a general sense of helplessness and perceived lack of control, this is postulated to trigger the onset of depression [7,19].

At present, several explanations of clinically relevant fatigue exist; however, all of these are restricted to specific disorders, such as diseases due to infections, inflammation, or autoimmune processes. For example, classical accounts of fatigue are mainly related to infectious disorders and the associated ‘sickness behavior’ (for a review, see [20]). Broader conceptualizations, with links to cognition, exist but are restricted to inflammatory conditions (for a review, see [21]). In addition, concepts of fatigue have been proposed for other specific disorders; for example, Heitmann et al. (2022; Ref. [22]) interpreted fatigue in multiple sclerosis as the consequence of inflammation-induced reduction in monoaminergic transmission and subsequent impairment of reward processing. By contrast, so far, the ASE theory is arguably the only general concept of fatigue that explains its ubiquitous occurrence across chronic disorders (with fundamental differences in the underlying pathophysiology) and combines biological, cognitive, and computational (algorithmic) perspectives. The theory offers testable predictions based on either (i) computational quantities (prediction error or surprise) which can be estimated from behavioral and/or neurophysiological data or on (ii) self-reported data about perceived control over bodily states (metacognition of allostatic control). In this study, we focus on the latter option.

Empirically, there is initial evidence that metacognition of allostatic control—as measured by a self-report questionnaire—is inversely associated with fatigue, as predicted by ASE theory [19]. However, a comprehensive investigation of the predictions made by the ASE theory is still lacking to date. Furthermore, as with almost all disease concepts in psychiatry, ASE theory has been formulated verbally, but not as a precise causal model.

Here, we present an initial attempt to tackle the latter issue. To this end, we identify variables and causal relations that are part of the ASE theory, namely metacognition of allostatic control (*M*; specifically, the feeling of being in control over one’s own bodily states), fatigue (*F*), general self-efficacy (*S*), and depression (*D*). We then formalize the causal structure implied by the ASE theory in the language of causal inference, more precisely, in the form of a structural causal model (SCM; Refs. [23,24,25]). Notably, the proposed SCM only contains variables and relations that are explicitly mentioned by the ASE theory in its current formulation [7]. In contrast to classical probabilistic models, an SCM induces not only an observational distribution but also a set of so-called interventional distributions. In other words, an SCM predicts how a system reacts under interventions [26]. We made use of a publicly available empirical dataset to test key aspects of the structure of the proposed SCM. Moreover, we used established methods for the estimation of average causal effects, focusing on central aspects of the ASE theory.

## 2. Materials and Methods

### 2.1. Empirical Dataset

In this work, we used data from a previous study conducted at the Translational Neuromodeling Unit (TNU) Zurich, the perception of breathing in the human brain (PBIHB) study; a detailed description of the dataset can be found elsewhere [27]. It comprises behavioral, questionnaire, and neuroimaging data from 60 healthy individuals. The questionnaire data used for our analysis are freely available for download from the Zenodo open data repository at https://doi.org/10.5281/zenodo.10992529 (accessed on 12 December 2024). Participants completed a battery of psychological questionnaires assessing subjective affective measures, both general and breathing-specific subjective interoceptive beliefs, as well as measures of general positive and negative affect, resilience, self-efficacy, and fatigue.

For our analysis, we focused on the following measures as representations of the central quantities of the ASE theory:

- fatigue (*F*): Fatigue Severity Scale (FSS)
- general self-efficacy (*S*): General Self-Efficacy Scale (GSES)
- depression (*D*): Centre for Epidemiologic Studies Depression Scale (CES-D)
- metacognition of allostatic control (*M*): Sum of the subscales 3 (not worrying) and 8 (trusting) of the Multidimensional Assessment of Interoceptive Awareness (${\mathrm{MAIA}}_{3,8}$).

One important caveat is that, to our knowledge, there does not yet exist a measure specifically developed for the construct of *M* (metacognition of allostatic control, i.e., the feeling of being in control over one’s own bodily states). In this study, as a proxy measure, we used the sum of the subscales 3 and 8 of the MAIA questionnaire. These subscales reflect an individual’s tendency not to experience distress in response to bodily inputs signaling dyshomeostasis and to perceive the body as a safe place, respectively. The sum of these subscales was used in a previous study testing predictions from ASE theory [19] and may currently represent the best approximation to *M* that is easily applied in practice.

### 2.2. SCM of the ASE Theory

An SCM [23,25] over variables $X=\left[X_{1},...,X_{n}\right]$ comprises a set of structural equations and distributions of the noise variables (a formal definition of an SCM is provided in Appendix Definition A1). The structural equations, together with the noise distributions, induce the observational distribution $P_{X}$ as a simultaneous solution to the structural equations [26]. In addition to the observational distribution, an SCM induces interventional distributions. Each intervention denotes a scenario in which we fix a certain subset of the variables to a certain value, e.g., $P_{do(X_{1}:=x_{1})}$.

We restricted our formulation of an SCM of the ASE theory to variables and relations that the current formulation of the ASE theory [7] explicitly comments on. Additionally, we considered the effects of the variables age (*A*) and gender (*G*), which are not directly part of the ASE theory, but are known to be associated with several variables of the SCM and may act as confounders. Under assumptions of linearity and normality, the SCM of the ASE theory takes the following form:

(1) $$\begin{array}{cc} A & =N_{a} \end{array}$$

(2) $$\begin{array}{cc} G & =N_{g} \end{array}$$

(3) $$\begin{array}{cc} \;M & =\theta_{1}A+\theta_{2}G+N_{m} \end{array}$$

(4) $$\begin{array}{cc} \;F & =\theta_{3}M+\theta_{4}A+\theta_{5}G+N_{f} \end{array}$$

(5) $$\begin{array}{cc} \;S & =\theta_{6}A+\theta_{7}G+N_{s} \end{array}$$

(6) $$\begin{array}{cc} \;D & =\theta_{8}F+\theta_{9}S+\theta_{10}FS+\theta_{11}A+\theta_{12}G+N_{d} \end{array}$$

where *A* stands for age, *G* for gender, *M* for metacognition of allostatic control, *F* for fatigue, *S* for general self-efficacy, and *D* for depression, and where $N_{i}$ are jointly independent noise variables. ∀i≠g, $N_{i}$ follows a normal distribution and $N_{g}$ is a Bernoulli random variable.

**Figure 1:**
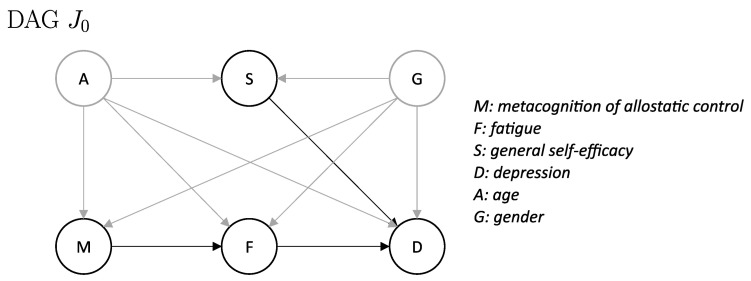
Directed acyclic graph (DAG) $J_{0}$ summarizing the key proposal of the allostatic self-efficacy theory (ASE; Ref. [7]). The DAG $J_{0}$ is representative of the induced observational distribution P and the interventional distributions induced by interventions on metacognition of allostatic control (*M*; $P_{do(M:=m)}$), fatigue (*F*; $P_{do(F:=f)}$), or general self-efficacy (*S*; $P_{do(S:=s)}$). The other variables in the graph are depression (*D*), age (*A*), and gender (*G*). Black edges represent causal directions as proposed explicitly by the ASE theory, grey edges represent effects from age and gender that are not explicitly part of the ASE theory but are included to account for potentially confounding effects.

Figure 1 displays a graphical summary of the causal structure implied by the ASE theory in the form of a directed acyclic graph (DAG) $J_{0}$. The directed edge from metacognition of allostatic control (*M*) to fatigue (*F*) represents the prediction that fatigue arises as a consequence of a metacognitive diagnosis by the brain—i.e., the brain concludes that it has low control over its bodily states. When this low allostatic self-efficacy (for which fatigue is the accompanying feeling state) is combined with beliefs of lack of control in other domains than the body (low general self-efficacy), this is predicted to lead to the onset of depression. These effects are represented by the directed edges from fatigue (*F*) to depression (*D*) and from general self-efficacy (*S*) to depression (*D*). The DAG $J_{0}$ in Figure 1 is representative of the induced observational distribution P and the interventional distributions induced by interventions on metacognition of allostatic control (*M*; $P_{do(M:=m)}$), fatigue (*F*; $P_{do(F:=f)}$), or general self-efficacy (*S*; $P_{do(S:=s)}$).

When taking a closer look at the causal graph in Figure 1, there are a number of points worth highlighting. (i) There is no direct link between metacognition of allostatic control (*M*) and general self-efficacy (*S*). (ii) There is no direct link between metacognition of allostatic control (*M*) and depression (*D*). All of its influence is mediated by fatigue (*F*). (iii) There is no direct link between fatigue (*F*) and general self-efficacy (*S*). While these three links are, in principle, plausible causal influences, they were not included in the original formulation of the ASE theory [7]. Whether these links should be included in a revision of the ASE theory can, in principle, be tested using methods of causal inference, given appropriate readouts of the involved quantities and relying on the assumption of the Markov condition.

### 2.3. Statistical Analysis

Our hypotheses, as well as the entire analysis, were pre-registered in a time-stamped analysis plan that is publicly available on the Zenodo open data repository at https://doi.org/10.5281/zenodo.10559656 (accessed on 12 December 2024). Below, we explicitly highlight any deviations from the pre-specified analysis plan. The analysis code is available at https://github.com/alexjhess/pbihb-ase-causality (accessed on 12 December 2024). The analysis pipeline underwent an internal code review by a researcher not involved in the initial data analysis to identify errors and ensure the reproducibility of our results.

#### 2.3.1. Causal Structure of ASE Theory in the PBIHB Dataset

Learning causal structure from observational data is inherently difficult. One reason for this is the existence of models that are observationally but not interventionally equivalent [25,26,28,29]. This has several implications (e.g., see [26]), one of them being that, without assumptions, it is impossible to learn causal structures from observational data.

In graphical models, the Markov condition (see, e.g., [30]) is a formalization of the following principle (sometimes referred to as Reichenbach’s common cause principle): If two random variables *X* and *Y* are dependent, then there must be some cause–effect structure that explains the observed dependence. That is, either *X* causes *Y*, or *Y* causes *X*, or another unobserved variable *H* causes both *X* and *Y*, or some combination of the aforementioned [31]. A formal definition of the Markov condition is presented in Appendix Definition A2. The Markov condition establishes a connection from graphical separation properties (*d*-separation; see Appendix Definition A3 for a formal definition) to conditional independencies in the distribution. Any distribution induced by an acyclic SCM satisfies the Markov condition with respect to the corresponding graph [25,32]. Hence, the Markov condition is typically considered to be a mild assumption.

Assuming that the observational distribution P induced by the SCM of the ASE theory (Equations (1)–(6)) is Markov with respect to the DAG $J_{0}$, we tested whether we found any contradictions to the structure of the DAG $J_{0}$ in the PBIHB dataset. More precisely, we examined the three predictions described in the last paragraph of Section 2.2 and formalized them as part of our pre-registered Hypothesis 1: Data from the PBIHB study satisfy the following conditional independence statements:

(i) M⫫S∣A,G
(ii) M⫫D∣F,A,G and M⫫D∣F,A,G,S
(iii) F⫫S∣A,G and F⫫S∣A,G,M

As a statistical test for conditional independence, we used an asymptotic $\chi^{2}$ test on the mutual information for conditional Gaussians (${\mathrm{MI}}_{cg}$) for mixed discrete and normal variables, as implemented in the R package bnlearn [33], using a significance level α=0.01 (Bonferroni corrected).

Since conditional independence testing is a difficult statistical problem [34], we validated our results using two alternative methods: a kernel conditional independence test (KCI; Ref. [35]) as implemented in the R package CondIndTests, and a test based on the generalized covariance measure (GCM; Ref. [34]), as implemented in the R package GeneralisedCovarianceMeasure. These additional tests of conditional independence were not part of our pre-specified analysis. We decided to conduct these additional tests to evaluate the robustness of our results across different methods of conditional independence testing (i.e., a sensitivity analysis). We used the same significance level α=0.01 for the KCI- and the GCM-based tests to ensure compatibility with the pre-specified tests.

#### 2.3.2. Estimating the Average Causal Effect from *M* to *F*

ASE theory predicts that fatigue is a feeling state that is triggered by a metacognitive diagnosis of loss of control over bodily states. We aimed to test this prediction as part of our Hypothesis 2: There is a negative average causal effect from metacognition of allostatic control (*M*) to fatigue (*F*)

(7) $$\frac{\partial }{\partial m}E_{do(M:=m)}\left[F\right]=\theta_{3}.$$

Adjusting for covariates is one of the various methods for estimating causal effects from observational data. Suppose we are interested in finding the effect of *M* on *F* and assume the factors deemed relevant to the problem are structured as in Figure 1. In other words, we are interested in calculating the intervention distribution $P_{do(M:=m)}\left(f\right)$. Given a valid adjustment set (VAS) Z, here e.g., Z=(A,G), the intervention distribution can be calculated (see [36,37,38]) as $P_{do(M:=m)}\left(f\right)=\sum_{z}P\left(f|m,z\right)P\left(z\right)$, since

(8) $$\begin{array}{cc} P_{do(M:=m)}\left(f\right) & =\sum_{z}P_{do(M:=m)}\left(f,m,z\right) \end{array}$$

(9) $$\begin{array}{cc} \; & \;=\sum_{z}P_{do(M:=m)}\left(f|m,z\right)P_{do(M:=m)}\left(m,z\right) \end{array}$$

(10) $$\begin{array}{cc} \; & \;=\sum_{z}P_{do(M:=m)}\left(f|m,z\right)P_{do(M:=m)}\left(z\right) \end{array}$$

(11) $$\begin{array}{cc} \; & \;=\sum_{z}P\left(f|m,z\right)P\left(z\right) \end{array}$$

where, in the last step, one can use the fact that causal relationships are autonomous under interventions (this property is sometimes referred to as ’autonomy’) [28].

In linear Gaussian systems, a causal effect from *M* to *F* can be approximated by $\frac{\partial }{\partial m}E_{do(M:=m)}\left[F\right]$ (see e.g., [28]). Assuming that Z is a VAS for {M,F} and {M,F},Z follow a Gaussian distribution, then the conditional F∣M=m,Z=z follows a Gaussian distribution as well. Hence, the mean of the distribution is given by

(12) $$E\left[F|M=m,Z=z\right]=\theta_{3}m+b^{t}z$$

for some $\theta_{3}$ and **b**. Equation (7) then follows from Equation (11).

One can estimate the conditional mean (Equation (12)) by regressing *F* on *M* and Z and subsequently reading off the regression coefficients for *M*. Alternatively, more sophisticated techniques for estimation of the average causal effect can be used, such as the propensity score method [39] and double/debiased machine learning (DML; Ref. [40]). In Appendix B, the two methods are described in more detail.

As pre-specified in our analysis plan, we conducted linear regression in combination with a one-sided *t*-test on the regression coefficient of *M* to evaluate Hypothesis 2. We compared our estimate of the causal effect from *M* to *F* obtained via linear regression with the results obtained from using more sophisticated estimation techniques, i.e., the propensity score method [39] and DML [40], following our pre-registered analysis plan.

#### 2.3.3. Estimating the Average Causal Effect from F*S on *D*

Another prediction of ASE theory is that fatigue, in combination with a generalization of low self-efficacy beliefs beyond bodily control, induces depression. We formalized this prediction as part of our Hypothesis 3: There is a negative average causal effect of the interaction term between fatigue and general self-efficacy (F*S) on depression (*D*)

(13) $$\frac{\partial }{\partial f\partial s}E_{do(F:=f,S:=s)}\left[D\right]=\theta_{10}.$$

Evaluation of Hypothesis 3 followed the same line of reasoning as for Hypothesis 2. We used linear regression in combination with a one-sided *t*-test on the regression coefficient of F*S. Subsequently, we compared the resulting estimate to the results obtained using the propensity score method and DML.

## 3. Results

### 3.1. Raw Data

**Figure 2:**
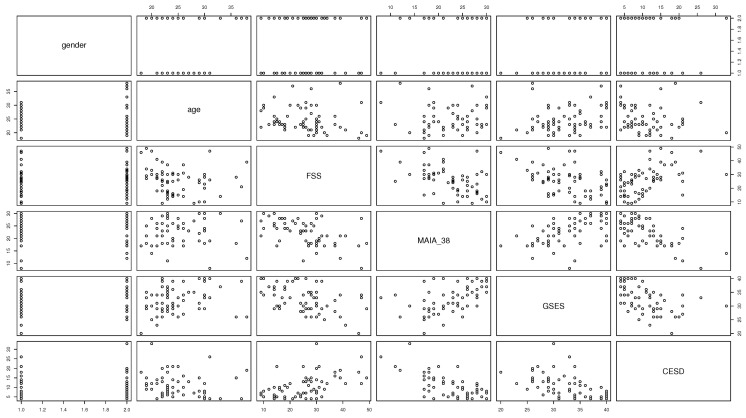
Scatter plot matrix of raw data used in the analysis. Displayed are all the pairwise scatter plots of the variables used for the analysis in a matrix format. For example, the scatter plot located at the intersection of row 3 and column 2 is a plot of the variables age versus fatigue (as measured by the FSS). The variables displayed are gender, age, fatigue (assessed by the FSS), metacognition of allostatic control (assessed by the ${\mathrm{MAIA}}_{3,8}$), self-efficacy (assessed by the GSES), and depression (assessed by the CES-D).

Figure 2 shows a scatter plot matrix of the raw data. Displayed are the measures for all variables A,G,M,F,S,D used in the analysis.

### 3.2. Results from the Statistical Analysis

#### 3.2.1. Causal Structure of ASE Theory in the PBIHB Dataset

Table 1 displays the results from conditional independence testing to evaluate the three predictions formulated as part of Hypothesis 1. The results can be summarized as follows:

(i) M⫫S∣A,G. We found significant evidence that metacognition of allostatic control (*M*) and general self-efficacy (*S*) are not independent conditional on age (*A*) and gender (*G*) across all three different conditional independence test methods. In other words, we found a contradiction between *d*-separation within the DAG $J_{0}$ and conditional independence of *M* and *S* given A,G.

(ii) M⫫D∣F,A,G and M⫫D∣F,A,G,S. We found significant evidence that *M* and depression (*D*) are not independent conditional on fatigue (*F*), *A*, *G* across all three methods for conditional independence testing. This result was consistent with our findings for (i), in the sense that if we add a directed edge from *M* to *S* in the DAG $J_{0}$ (Figure 1), the only set of variables that *d*-separates *M* and *D* is the set F,A,G,S (and not F,A,G). However, the results for conditional independence tests of *M* and *D* conditional on F,A,G,S were mixed, with 2 out of 3 tests (GCM and KCI) not reaching the pre-specified significance level α=0.01. Hence, further evidence is needed to draw conclusions regarding the statement M⫫D∣F,A,G,S.

(iii) F⫫S∣A,G and F⫫S∣A,G,M. When looking at the conditional independence between *F* and *S*, the results depended on the set of variables that we conditioned on. We found significant evidence that *F* and *S* are not independent conditional on A,G across all three different test methods. However, we failed to reject the null hypothesis that *F* and *S* are independent conditional on the set M,A,G consistently across all three different test methods. This result is also in line with our findings for (i), in the sense that if we add a directed edge from *M* to *S* in the DAG $J_{0}$ (Figure 1), the only set of variables that *d*-separates *F* and *S* is the set M,A,G.

#### 3.2.2. Estimating the Average Causal Effect from *M* to *F*

As predicted by the ASE theory, we found significant evidence for a negative average causal effect from metacognition of allostatic control (*M*) to fatigue (*F*) $\frac{\partial }{\partial m}E_{do(M:=m)}\left[F\right]=\theta_{3}$ across all three different estimation methods. The resulting estimates ${\hat{\theta }}_{3}$ for the VAS Z=(A,G) are displayed in Table 2, alongside lower and upper bounds of a 95% confidence interval for ${\hat{\theta }}_{3}$, the corresponding value of the *t*-statistic, as well as the *p*-value for the one-sided *t*-test.

The results from our sensitivity analysis, i.e., estimating $\theta_{3}$ using a different VAS Z=(A,G,S), are listed in Table 3. They confirmed the finding of a negative average causal effect from *M* to *F* when using Z=(A,G) as a VAS. The main difference between the results of the two analyses was that the second analysis using Z=(A,G,S) yielded a slightly lower absolute value for ${\hat{\theta }}_{3}$, as well as a non-significant *p*-value using the DML method.

#### 3.2.3. Estimating the Average Causal Effect from F*S to *D*

We did not find evidence for the predicted negative average causal effect of the interaction term between fatigue and general self-efficacy (F*S) on depression (*D*) $\frac{\partial }{\partial f\partial s}E_{do(F:=f,S:=s)}\left[D\right]=\theta_{10}$ across all three different estimation methods for either VAS Z=(A,G) or Z=(A,G,M). Tables containing the resulting estimates for ${\hat{\theta }}_{10}$ including a 95% confidence interval and the value of the *t*-statistic, as well as the *p*-value for the one-sided *t*-test, are listed in Appendix C.

## 4. Discussion

In this paper, we proposed a formulation of the allostatic self-efficacy (ASE) theory of fatigue and depression in the language of causal inference. Specifically, we identified the variables of central interest to the ASE theory and formulated a structural causal model (SCM) under assumptions of linearity and normality. The SCM, as well as the induced directed acyclic graph (DAG), describe the direction of causality among these variables. The proposed SCM only contains variables and relations that were explicitly suggested by the ASE theory (together with age and gender as potential confounders) in its current formulation [7]. This is not meant to imply that other variables or connections cannot be present or may not be included in future revisions of the theory. However, in this preregistered analysis, the focus was entirely on the ASE theory in its current form. Using the data of 60 healthy individuals from a previous study on interoception of breathing and its relation with several psychopathological constructs [27], we tested the proposed causal model empirically. Relying on the assumption of the Markov condition, we used the dataset to search for contradictions to conditional independence statements (Hypothesis 1) that are implied by the graph structure (*d*-separation). In a second and third step, we estimated the value of two causal effects that are predicted by the ASE theory using methods of covariate adjustment, propensity scores, and double/debiased machine learning. As predicted by the ASE theory, we found a statistically significant negative average causal effect from metacognition of allostatic control (*M*) to fatigue (*F*) $\frac{\partial }{\partial m}E_{do(M:=m)}\left[F\right]=\theta_{3}$ across all three methods of estimation. Our sensitivity analysis using a different valid adjustment set largely confirmed this finding with two out of three estimation methods yielding a significant result.

The assumption of the Markov condition establishes a connection from *d*-separation statements in a causal graph to conditional independence statements in the distribution. In the analysis of Hypothesis 1, we tested concrete predictions implied by the DAG $J_{0}$ (Figure 1). (i) Using the data from the PBIHB study, we were able to reject the null hypothesis of M⫫S∣A,G at the pre-specified level α=0.01. (ii) We found significant evidence against M⫫D∣F,A,G in the empirical dataset. However, in line with the graph structure $J_{0}$ implied by the ASE theory, we did not find clear evidence against M⫫D∣F,A,G,S. That is, only one out of three conditional independence tests rejected the null hypothesis of metacognition of allostatic control (*M*) being independent from depression (*D*) conditional on the set F,A,G,S. (iii) We also found significant evidence against F⫫S∣A,G in the empirical data. Yet, we did not find any evidence against F⫫S∣A,G,M. All three conditional independence test methods consistently failed to reject the null hypothesis of fatigue (*F*) and general self-efficacy (*S*) being independent given the set A,G,D,M.

There are a number of potential explanations for the results related to Hypothesis 1. The most straightforward explanation is that the proposed causal model is incorrect. This could include the presence of additional edges between nodes, as well as variables that were not considered, acting as mediators or confounds or a combination of all of the aforementioned. For example, although ASE theory does not make an explicit statement about a direct link between metacognition of allostatic control (*M*) and general self-efficacy (*S*), it is plausible to assume the existence of a directed edge from *M* (the feeling of control over bodily states) to *S* (an individual’s general expectation of personal mastery and control [41]). The construct of *S* is closely related to concepts of metacognition (see, e.g., [42]) and represents a ’global’ construct of self-beliefs about one’s capacity to achieve goals and overcome adversity; this can be understood as including more ’local’ domain-specific forms of self-efficacy, such as metacognition of allostatic control. From this viewpoint, the idea that metacognition of allostatic control (*M*) may contribute to (and thus influence) general self-efficacy (*S*) beliefs is therefore not entirely unreasonable and would be a potential explanation for the results of (i) and (iii). More precisely, a directed edge from *M* to *S* would render *M* and *S d*-connected, since there would always exist a path between *M* and *S* that is not blocked by any set of variables. This cause–effect structure would explain the observed dependence between the two variables in the empirical dataset according to Reichenbach’s common cause principle [31]. Another consequence of introducing an edge from *M* to *S* would be that the set of variables that *d*-separates *F* and *S* would consist of variables A,G,M and not only A,G, which corresponds to our findings for (iii). The same is true for the set of variables *d*-separating *M* and *D*, which would consist of variables F,A,G,S and not F,A,G in this case, potentially explaining our findings for (ii). However, since the evidence for M⫫D∣F,A,G,S was mixed, further research is needed to bring clarity to the question of the (conditional) independence of *M* and *D*.

**Figure 3:**
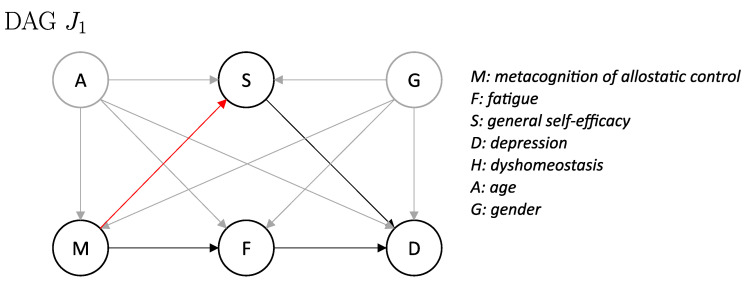
Updated directed acyclic graph (DAG) $J_{1}$ of the allostatic self-efficacy theory (ASE; Ref. [7]) providing a potential explanation for the observed results from analysis of Hypothesis 1. Modifications from DAG $J_{0}$ to $J_{1}$ are shown in red.

The revised DAG $J_{1}$ (Figure 3) provides a graphical summary of the above considerations regarding the results related to Hypothesis 1. From DAG $J_{0}$ to $J_{1}$, we added a directed edge from *M* to *S*. However, there are several other potential explanations for the observed results, so this example should by no means be taken as ’the correct model’. If anything, this should be regarded as an updated hypothesis to be tested in future investigations.

One of our reviewers wondered why we did not compare different graph structures against each other by evaluating how well they fit the empirical data. In causal inference, this approach would belong to the class of score-based methods for causal discovery [28]. This is certainly an interesting alternative strategy, provided a suitable generative model exists, and may well be adopted in future work on this topic. By contrast, the approach in this work belongs to the category of independence-based methods for causal structure learning. It consists of specifying a theory in the form of an SCM and subsequently testing which parts of the associated DAG $J_{0}$ are inconsistent with empirical data using conditional independence tests, which subsequently leads to an amended DAG $J_{1}$. This was particularly well suited for our problem, since the DAG $J_{0}$ was based only on the variables and relations explicitly suggested by ASE theory [7] that we wanted to examine and refine.

Concerning Hypothesis 2, we found evidence for a negative average causal effect from metacognition of allostatic control (*M*) to fatigue (*F*) $\frac{\partial }{\partial m}E_{do(M:=m)}\left[F\right]=\theta_{3}$ across all three estimation methods (covariate adjustment, propensity scores, DML) for two different VAS. This is in line with the prediction by ASE theory that the subjective experience of fatigue arises as a consequence of a metacognitive diagnosis that the brain’s control over bodily states is failing (low allostatic control). This also confirms findings from previous research, which identified metacognition of allostatic control (*M*) (operationalized by the sum of the subscales 3 and 8 of the MAIA questionnaire) as associated with fatigue (*F*) scores [19]. Our new results go beyond this previous finding, in the sense that the current study suggests a direction of the effect, as opposed to purely associative statements. It is worth highlighting that the estimation of the causal effect from *M* to *F* would not be affected by the proposed additional link between *M* to *S* as suggested by the analysis results concerning Hypothesis 1 (see Figure 3), since the set A,G would still be a valid adjustment set (VAS).

With regard to Hypothesis 3, we did not find evidence for a negative average causal effect of the interaction term between fatigue and general self-efficacy (F*S) on depression (*D*) $\frac{\partial }{\partial f\partial s}E_{do(F:=f,S:=s)}\left[D\right]=\theta_{10}$. The present work is, to the best of our knowledge, the first attempt to investigate the predicted influence of the interaction between fatigue and general self-efficacy on depression. Across all three different estimation methods and using different VAS, we found, if anything, very small effects. However, one may rightfully question whether the sample in this study was adequate for testing Hypothesis 3, at least in the context of the ASE theory. This is because our participants were drawn from the general population and, not surprisingly, did not show pronounced levels of depression (compare Figure 2). By contrast, the predictions of ASE theory concerning depression assume a clinically relevant state of depression [7]. Therefore, the potential interaction effect F*S on *D* remains an open question that should be addressed in the future using samples with clinically relevant levels of depression. In the context of depression, it is worth mentioning that the current formulation of the ASE theory does not make any explicit reference to emotional states. This could potentially be addressed in future developments by extending the generic Bayesian model of brain–body interactions that represents the foundation of the ASE theory [7] with developments from active inference which introduce explicit representations of valence (e.g., [43]).

One might ask why we did not use a more complex causal model and examine any potential bidirectional causal effects between the variables of interest, e.g., fatigue and depression. Indeed, the lack of bidirectional causal influences in our model can be regarded as a limitation, in that it prevents identification of an optimal causal structure from data without constraints. However, the objective of the current study was to examine whether the causal structure implied by a particular theory (i.e., ASE) is compatible with available cross-sectional questionnaire data. Notably, the complexity of the proposed SCM was commensurate with both the level of complexity of the ASE theory and the cross-sectional dataset, i.e., our model only contains those variables and relations that are explicitly suggested by the ASE theory in its current formulation [7] and were measured using available questionnaires. Nevertheless, should future formulations of the ASE theory include bidirectional effects, the current methodological approach (i.e., conditional independence tests in the context of DAGs) could be extended. Specifically, if longitudinal timeseries data are available, DAGs can be extended in time, which allows testing cyclic and bidirectional causal effects (for examples, see [44,45]), thus addressing the limitation discussed above. Furthermore, if required by the theory to be tested, longitudinal data would also enable one to consider the role of time. While not required for the purpose of the current study (since the current formulation of ASE theory does not make any statements about the temporal dynamics of variables and their relations), a more exploratory approach could examine, for example, whether causal influences operate at particular time scales. Such putative extensions could employ other methods of causal inference, e.g., Granger causality [46] convergent cross mapping [47], or dynamic causal modeling (e.g., [48]), once longitudinal timeseries data for the variables of interest become available.

The current study has strengths and limitations. Its strengths include the first concrete formulation of the ASE theory in the language of causal inference: our proposal of an SCM brings the content of a verbally formulated theory into the realm of concrete mathematical equations. Together with the induced DAG, this provided a formal basis for analysis and allowed us to identify a set of empirically testable hypotheses which may guide future research. A second notable strength is that we used multiple independent methods, both for conditional independence testing (Hypothesis 1) and for the estimation of causal effects (Hypotheses 2 and 3). This enhances the robustness of our conclusions, since they do not depend on assumptions and properties of any single method. Last but not least, all of our hypotheses and statistical analysis procedures were pre-registered and specified in detail in an ex ante analysis plan (https://doi.org/10.5281/zenodo.10559656 (accessed on 12 December 2024)). Preregistration is an important and effective protection for the robustness of research, given the many degrees of freedom and the numerous cognitive biases that scientists may inadvertently be affected by [49].

Turning to limitations, first, as in any observational study, one may question whether the results may have been affected by (unknown or non-measured) confounding variables (compare our discussion of the results from Hypothesis 1 above). Specifically, one limitation is that our current analysis did not take sleep into account. While sleep is not an explicit component of the ASE theory, previous work has repeatedly demonstrated that sleep quality generally influences the level of fatigue (e.g., [19,50]). In the present study, we did not examine the potential influence of sleep, since the available dataset did not include any measures of sleep quality. This will be rectified in follow-up studies.

Second, our statistical approach made a number of assumptions, as explained in the Methods. Most of these were standard assumptions and not specific to our study, but are still worth keeping in mind, since violation of these assumptions may affect the results. For example, our structural model assumed that effects are linear and random variables (except gender) follow a normal distribution. Furthermore, as already discussed above, our statistical approach did not take into account bidirectional causal interactions. This is because our causal model is constrained by the explicit assumptions of the ASE theory (which does not include bidirectional causal effects). Finally, conditional independence testing, which is at the heart of causal discovery [38], is generally challenging [51]; we, therefore, employed multiple independent tests to ensure our results were robust.

Finally, our dataset has several properties that deserve consideration. To begin with, our dataset is cross-sectional and observational, which is the norm for studies in neuroscience but challenging for causal inference. To facilitate causal inference, follow-up studies could include interventions (e.g., cognitive interventions targeting *M*) and/or acquire longitudinal data. Another issue is the question of sample size. While the size of our sample (N = 60) was large for the neuroscientific focus of the original study, it was not determined in advance for the questions addressed in the current study. Furthermore, the question of what constitutes adequate sample sizes for causal inference from observational data is complex and controversial [52]. It is therefore important to replicate our findings in independent (and larger) samples. A third issue concerns the validity of the available measurements. While validated self-report measures for fatigue, depression, and general self-efficacy are available and were used in this study, a measurement tool specifically developed for the construct of metacognition of allostatic control (*M*) is lacking so far. Here, we followed previous research [19] and used a plausible proxy measure of *M*, i.e., the sum of two pre-specified subscales from a validated questionnaire on interoception, MAIA. An important goal for future research is the development of readouts for *M* that are fully validated and easily applied in practice. Ideally, such tests would go beyond self-report measures and be implicit in nature, e.g., neurophysiological readouts similar to the mismatch negativity.

## 5. Conclusions

In summary, our work provides a formal basis for testing predictions by the ASE theory of fatigue and depression in the context of causal inference. We evaluated central aspects of our proposed SCM using a publicly available dataset and provided an updated version of the SCM that accounts for our empirical findings. In addition, we were able to confirm previous findings regarding the association between metacognition of allostatic control (*M*) and fatigue (*F*). Our analysis enabled us to quantify the direction, as well as the sign, of the causal effect, i.e., we found a negative average causal effect from *M* to *F* $\frac{\partial }{\partial m}E_{do(M:=m)}\left[F\right]=\theta_{3}$, as predicted by the ASE theory. Finally, we identified a number of open questions that remain to be addressed in future research and that may help unravel the mechanisms behind fatigue and depression.

## Figures and Tables

**Table 1:** Results from different conditional independence test methods (${\mathrm{MI}}_{cg}$, GCM, KCI) for the three predictions formulated as part of Hypothesis 1. Results are presented for three different test methods. An asterisk indicates statistically significant evidence against the null hypothesis ($H_{0}$: variables are conditionally independent) using the pre-specified level α=0.01, which corresponds to a threshold of p<0.05 Bonferroni corrected for the multiple comparisons of the five tests, *p*-values are shown in parentheses.

Hypothesis 1	*d*-Separation Statement	${\mathrm{MI}}_{\mathrm{cg}}$ (*p*-Value)	GCM (*p*-Value)	KCI (*p*-Value)
(i)	$M{⫫}_{J_{0}}S|A,G$	22.044 * (1.634 × $10^{-5}$)	4.254 * (2.104 × $10^{-5}$)	26.451 * (5.194 × $10^{-6}$)
(ii)	$M{⫫}_{J_{0}}D|F,A,G$	24.167 * (5.652 × $10^{-6}$)	−3.131 * (0.001743)	8.513 * (0.001346)
	$M{⫫}_{J_{0}}D|F,A,G,S$	16.883 * (0.000216)	−2.574 (0.010064)	2.992 (0.022626)
(iii)	$F{⫫}_{J_{0}}S|A,G$	13.010 * (0.001496)	−3.390 * (0.000700)	13.613 * (0.001279)
	$F{⫫}_{J_{0}}S|A,G,M$	4.057 (0.131500)	−2.088 (0.036799)	2.013 (0.118908)

* *p* < 0.01.

**Table 2:** Average causal effect from *M* to *F* using Z=(A,G). Displayed are estimates of the average causal effect from *M* to *F* ${\hat{\theta }}_{3}$ across three different methods to adjust for the covariates Z=(A,G). We report a point estimate ${\hat{\theta }}_{3}$, the lower and upper bounds of a 95% confidence interval for ${\hat{\theta }}_{3}$, the value of the *t*-statistic, as well as the *p*-value for the one-sided *t*-test. An asterisk indicates statistical significance using the pre-specified level α=0.017 (Bonferroni-corrected).

Estimation Method	${\hat{\theta }}_{3}$	Confidence Interval		*t* Value	*p*-Value
linear regression	−0.4845 *	−0.712	−0.257	−4.259	3.968 × $10^{-5}$
propensity score	−0.4816 *	−0.717	−0.246	−4.092	6.689 × $10^{-5}$
DML	−0.3872 *	−0.6481	−0.1262	−2.9082	0.0018

* p<0.017.

**Table 3:** Average causal effect from *M* to *F* using Z=(A,G,S). Displayed are estimates of the average causal effect from *M* to *F* ${\hat{\theta }}_{3}$ across three different methods to adjust for the covariates Z=(A,G,S). We report a point estimate of ${\hat{\theta }}_{3}$, the lower and upper bounds of a 95% confidence interval for ${\hat{\theta }}_{3}$, the value of the *t*-statistic, as well as the *p*-value for the one-sided *t*-test. An asterisk indicates statistical significance using the pre-specified level α=0.017 (Bonferroni-corrected).

Estimation Method	${\hat{\theta }}_{3}$	Confidence Interval		*t* Value	*p*-Malue
linear regression	−0.3545 *	−0.610	−0.099	−2.785	0.0037
propensity score	−0.3775 *	−0.692	−0.063	−2.400	0.0098
DML	−0.2049	−0.563	0.153	−1.122	0.1309

* p<0.017.

## Data Availability

Data used in this study are publicly available for download from the Zenodo open data repository https://doi.org/10.5281/zenodo.10992529 (accessed on 12 December 2024). The statistical analysis plan detailing the analysis ex ante is available at https://doi.org/10.5281/zenodo.10559656 (accessed on 12 December 2024). The analysis code is available at https://github.com/alexjhess/pbihb-ase-causality (accessed on 12 December 2024).

## Abbreviations

The following abbreviations are used in this manuscript:

MDPI	Multidisciplinary Digital Publishing Institute
ASE	allostatic self-efficacy
SCM	structural causal model
DAG	directed acyclic graph
ICD-10	International Statistical Classification of Diseases and Related Health Problems, 10th revision
DSM-5	Diagnostic and Statistical Manual of Mental Disorders, Fifth Edition
*A*	age
*G*	gender
*M*	metacognition of allostatic control
*F*	fatigue
*S*	general self-efficacy
*D*	depression
*N*	noise variable
*F***S*	the interaction term between fatigue and general self-efficacy
PBIHB	perception of breathing in the human brain
TNU	Translational Neuromodeling Unit
FSS	Fatigue Severity Scale
GSES	General Self-Efficacy Scale
CES-D	Centre for Epidemiologic Studies Depression Scale
MAIA	Multidimensional Assessment of Interoceptive Awareness
${\mathrm{MI}}_{cg}$	mutual information for conditional Gaussians
KCI	kernel conditional independence test
GCM	generalized covariance measure
DML	double/debiased machine learning
VAS	valid adjustment set

## Appendix A. Definitions

### Appendix A.1. Structural Causal Model

We adopt the definition of SCMs according to [26]:

**Definition A1.** 
*An SCM over variables $X=\left[X_{1},...,X_{n}\right]$ comprises*

- *structural equations which relate each variable $X_{k}$ to its parents $PA\left(X_{k}\right)\subseteq \left\{X_{1},...,X_{n}\right\}$ and a noise variable $N_{k}$ via a function $f_{k}$, such that $X_{k}:=f_{k}\left(PA\left(X_{k}\right),N_{k}\right)$, as well as a*
- *noise distribution $P_{N}$ of the noise variables $N={\left[N_{1},...,N_{n}\right]}^{T}$.*

*In a directed causal graph associated with an SCM, the nodes correspond to the variables $X_{1},...,X_{n}$ and there is an edge from $X_{i}$ to $X_{j}$ whenever $X_{i}$ appears on the right-hand side of the equation $X_{j}:=f_{j}\left(PA\left(X_{j}\right),N_{j}\right)$. In other words, if $X_{i}\in PA\left(X_{j}\right)$ the graph contains the edge $X_{i}\to X_{j}$. For this work, we assume that the graph does not contain any cycles. The structural equations together with the noise distributions induce the observational distribution $P_{X}$ of $X_{1},...,X_{n}$ as a simultaneous solution to the equations.*

### Appendix A.2. Markov Condition

**Definition A2.** 
*Given a DAG G over nodes X, we say that the distribution $P_{X}$ satisfies*

(i) *the **global Markov property** (MP) with respect to G if *∀* disjoint A,B,C⊆X*
  *A d-sep B∣C⇒A⫫B∣C*
(ii) *the **local Markov property** (MP) if $\forall j\;X_{j}⫫{\mathrm{ND}}_{j}\;|{\mathrm{PA}}_{j}$*
(iii) *the **factorization property** if $P_{X}$ is absolutely constant with respect to a product measure and $\forall x\forall j,\;p\left(x_{{\mathrm{PA}}_{j}}\right)>0:p\left(x\right)=p\left(x_{1},...,x_{d}\right)=\prod_{j}^{d}p\left(x_{j}|x_{{\mathrm{PA}}_{j}}\right)$*

In the above definition, we used the following notation: ${\mathrm{ND}}_{j}$ represents the non-descendants of node $X_{j}$ and ${\mathrm{PA}}_{j}$ denotes all nodes that have a directed edge to node $X_{j}$.

### Appendix A.3. d-Separation

**Definition A3.** 
*d-separation is a graphical criterion of whether two nodes are connected or not. Let X, Y, Z disjoint.*

(i) *A path $X=i_{1},...,i_{m}=Y$ is blocked by Z⇔*∃* node $i_{k}$ with $i_{k-1}\to i_{k}\to i_{k+1}$ and $i_{k}\in Z$*
  *OR *∃* node $i_{k}$ with $i_{k-1}\leftarrow i_{k}\leftarrow i_{k+1}$ and $i_{k}\in Z$*
  *OR *∃* node $i_{k}$ with $i_{k-1}\leftarrow i_{k}\to i_{k+1}$ and $i_{k}\in Z$*
  *OR *∃* node $i_{k}$ with $i_{k-1}\to i_{k}\leftarrow i_{k+1}$ and $i_{k}\notin Z$ and $DE(i_{k})\cap Z=\emptyset$*
(ii) *X, Y are d-connected given Z⇔∃X∈X,Y∈Y s.t. *∃* path between X and Y that is not blocked*
(iii) *if X, Y are not d-connected, then they are d-separated. We sometimes write X d-sep Y∣Z or $X{⫫}_{G}Y|Z$*

## Appendix B. Estimating Causal Effects Using Covariate Adjustment

### Appendix B.1. The ‘Propensity Score’ Method

In a point treatment situation, one can adjust for a set of confounders Z=(A,G) when estimating the effect of exposure *M* by weighting observations *i* by the inverse probability weights

(A1) $$w_{i}=\frac{1}{P(M_{i}=m_{i}|Z_{i}=z_{i})}$$

To increase statistical efficiency, one can use stabilized weights, e.g.,

(A2) $$sw_{i}=\frac{P(M_{i}=m_{i})}{P(M_{i}=m_{i}|Z_{i}=z_{i})}$$

When dealing with a continuous exposure variable *M*, one can use stabilized weights

(A3) $$sw_{i}=\frac{f(m_{i})}{f(m_{i}|z_{i})}$$

where $f(m_{i})$ is the marginal density function of *M*, evaluated at the observed value in unit *i*, $m_{i}$, and $f(m_{i}|z_{i})$ conditional density function of *M* given **Z**, evaluated at the observed values in unit *i*, $\left\{m_{i},z_{i}\right\}$ [53]. Weighting observations *i* by $sw_{i}$, one can fit a causal model, for instance a marginal structural model (MSM)

(A4) $$E\left[F_{m}\right]=\beta_{0}+\theta_{3}m$$

with continuous outcome fatigue *F*. The response variable $F_{m}$ is the potential outcome that could have been observed in a unit under study if that unit received a specific treatment level *m* [54]. The expectation $E\left[F_{m}\right]$ is the mean outcome if all units under study received a specific treatment level *m*. Parameter $\theta_{3}$ then quantifies the causal effect of *M* on *F* [53].

### Appendix B.2. Double/Debiased Machine Learning

DML removes the impact of regularization bias and overfitting on estimation of the parameter of interest $\theta_{3}$ by using Neyman-orthogonal moments and cross-fitting [40]. One application of DML is in the context of a partial linear regression model,

(A5) $$\begin{array}{cc} F=M\theta_{3}+k_{0}\left(Z\right)+N_{f} & ,\;E(N_{f}|M,Z)=0, \end{array}$$

(A6) $$\begin{array}{cc} M=m_{0}\left(Z\right)+V & ,\;E(V|Z)=0, \end{array}$$

with fatigue *F*, metacognition of allostatic control *M*, a VAS Z=(A,G) consisting of confounding covariates and stochastic error terms $N_{f}$ and *V*. The confounding covariates **Z** affect *M* and *F* via the functions $m_{0}$ and $k_{0}$, respectively. DML can be used to estimate $\theta_{3}$, i.e., the main regression coefficient that we would like to infer, which can be interpreted as the average causal effect from *M* to *F* [55].

## Appendix C. Results from Estimating the Average Causal Effect from F*S to D

**Table A1:** Average causal effect of the interaction term *F***S* to *D* using Z=(A,G). Displayed are estimates of the average causal effect of the interaction term *F***S* to *D* ${\hat{\theta }}_{10}$ across three different methods to adjust for the covariates Z=(A,G). We report a point estimate of ${\hat{\theta }}_{10}$, the lower and upper bounds of a 95% confidence interval for ${\hat{\theta }}_{10}$, the value of the *t*-statistic, as well as the *p*-value for the one-sided *t*-test.

Estimation Method	${\hat{\theta }}_{10}$	Confidence Interval		*t* Value	*p*-Value
linear regression	0.0281	−0.191	0.247	0.257	0.6010
propensity score	0.0142	−0.151	0.180	0.172	0.5680
DML	−0.2051	−0.476	0.066	−1.482	0.0691

**Table A2:** Average causal effect of the interaction term *F***S* to *D* using Z=(A,G,M). Displayed are estimates of the average causal effect of the interaction term *F***S* to *D* ${\hat{\theta }}_{10}$ across three different methods to adjust for the covariates Z=(A,G,M). We report a point estimate of ${\hat{\theta }}_{10}$, the lower and upper bounds of a 95% confidence interval for ${\hat{\theta }}_{10}$, the value of the *t*-statistic, as well as the *p*-value for the one-sided *t*-test.

Estimation Method	${\hat{\theta }}_{10}$	Confidence Interval		*t* Value	*p*-Value
linear regression	0.0671	−0.122	0.257	0.711	0.7599
propensity score	0.0337	−0.159	0.227	0.350	0.6362
DML	0.0153	−0.283	0.314	0.100	0.5399
